# Fatal pulmonary thromboembolism associated with uterine carcinosarcoma

**DOI:** 10.4322/acr.2023.438

**Published:** 2023-07-13

**Authors:** Giorgia Lodetti, Stefano Tambuzzi, Salvatore Andreola, Guendalina Gentile, Riccardo Zoja

**Affiliations:** 1 University of Milan, Department of Biomedical Sciences for Health, Laboratory of Forensic Histopathology and Microbiology - Section of Forensic and Insurance Medicine, Milan, Italy

Uterine carcinosarcoma (USC), also known as malignant mixed Müllerian tumor, is a rare and high-grade endometrial cancer composed of epithelial and mesenchymal elements.^1^ UCS accounts for 4.3% of all uterine corpus cancer, with a worldwide annual incidence of 0.5-3.3 cases per 100.000 women.^1^ Common risk factors include obesity, nulliparity, chronic endogenous or exogenous hyperestrogenism exposure, older age (with a median age of 70 years), African-American ethnicity, genetic predisposition, tamoxifen therapy, and prior pelvic irradiation.^2^

Typical UCS-related symptoms, unfortunately, associated with an already advanced stage of the disease, are large masses occupying the abdominal cavity, postmenopausal metrorrhagia, and abdominal pain. One of the most important complications of endometrial tumors, especially in UCS, is venous thromboembolism (VTE), with an incidence of 8.1%.^3-4^ It may also involve pulmonary arteries and is usually associated with aggressive tumor characteristics and poor patient health.^3-4^

While Trans-Vaginal Sonography (TVS) is the first-line method for the assessment of UCS, as it can detect neoformations of the myometrium, diagnosis can usually be made by the histopathological examination of targeted endometrial biopsies, possibly associated with hysteroscopy.^5-6^ Magnetic resonance imaging (MRI) is the best imaging modality for staging. Furthermore, it shows a typical high enhancement with a “spotty” distribution, which may suggest bleeding and is a distinct hallmark of carcinosarcoma.^6-7^

Depending on the histopathologic assessment of the tumor (depth of invasion and presence or absence of metastases) and clinical presentation,^8^ the management of UCS requires a multimodality treatment comprised of surgery, systemic therapy, and radiotherapy. The estimated 5-year survival rate ranges from 33 to 39%, but the recurrence rate remains high.^2,8^

**Figure 1:**
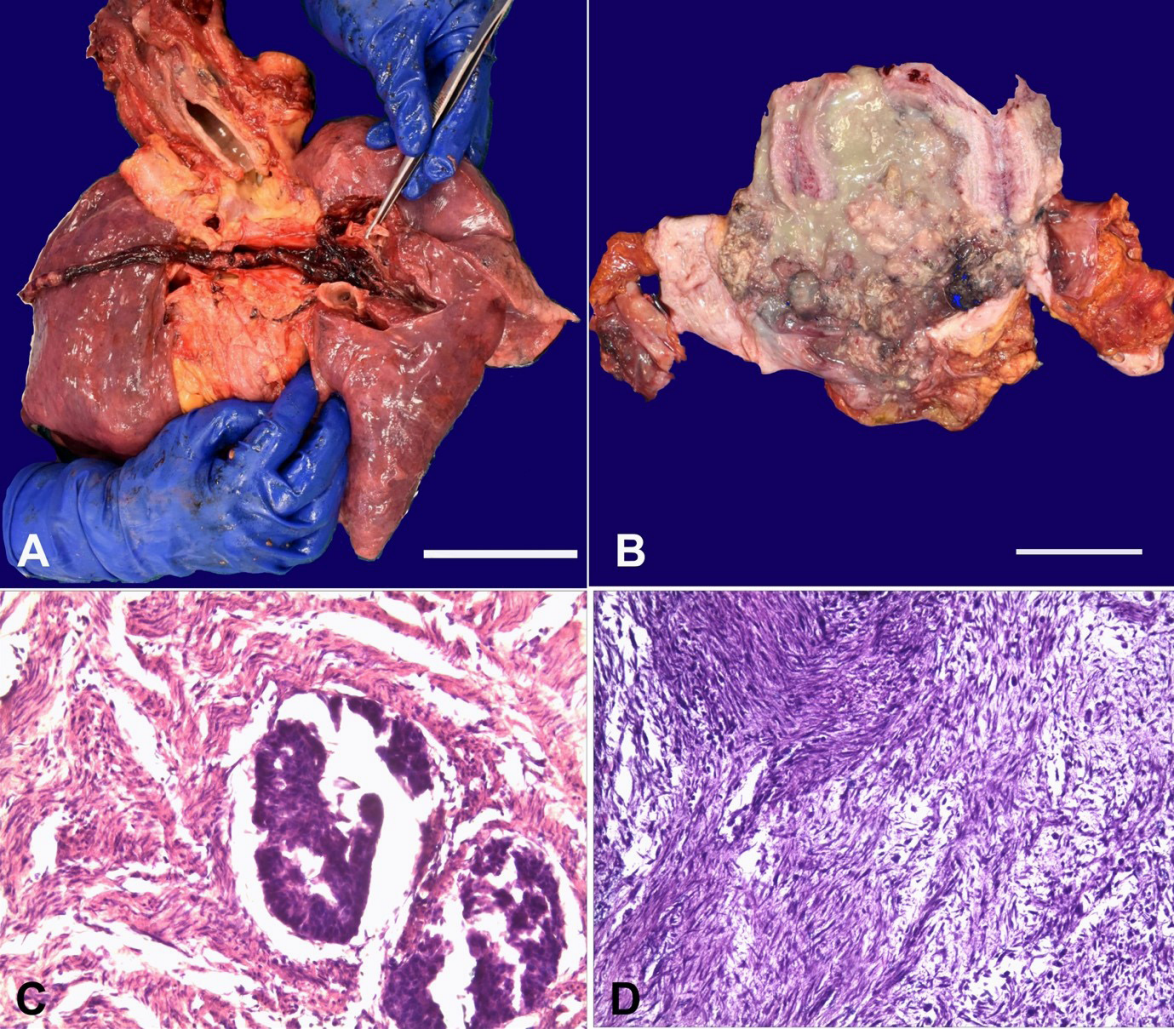
**A -** Macroscopic view of lungs showing red-brownish clots in the pulmonary artery trunk extending along the intraparenchymal branches (scale bar= 8 cm); **B -** Macroscopic view of the uterus after dissection with a vegetative neoformation almost occluding its lumen, in association with abundant yellow-green-blackish material of pasty consistency (scale bar= 7 cm); **C -** Photomicrograph of the endometrial tumor with remnants of neoplastic glandular epithelial component and of residual glandular pseudo-lumen with multi-layered epithelium and cellular atypia (H&E, 200x); **D -** Photomicrograph of the endometrial tumor with sarcomatous component partly with fused cellularity, partly in the form of compact bundles with marked nuclear atypia and partly with a myxoid-like structure with foci arranged in bundles associated with a scattered cell neoplastic component (H&E; 100x).

Figure 1 refers to a 61-year-old woman found dead in her flat, dressed in a sanitary napkin and a nightgown. Because the woman lived alone and had no family members, no health information could be achieved. An autopsy was performed to determine the cause of death. The body was in a good state of preservation; it weighed 85 kg and was 158 cm long (BMI = 34 Kg/m2 - Class I obesity). The sanitary napkin was smeared with a yellow-green-blackish creamy material upon external examination,. At dissection, both branches of the pulmonary artery trunk were occupied by red-brownish clots with multiple whitish streaks extending along the intraparenchymal course of the dividing branches of the pulmonary arteries (Figure 1A). They appeared to adhere tenaciously to the intimal surface, replicating the shape of the vascular lumen as a mold. All these characteristics were confirmed upon slicing, and a friable consistency was noted. On the abdominal incision, abundant yellow-green-blackish material of pasty consistency and purulent appearance was noted near the pelvic notch and between some intestinal loops. The uterus (longitudinal diameter = 18 cm, transversal = 11 cm, anteroposterior = 5 cm) appeared to be increased in volume, and the wall had a wall thickness of 1.5 cm. Inside the uterine cavity, a vegetative neoformation was detected (longitudinal diameter = 6 cm, transversal = 5 cm, anteroposterior = 4 cm), almost occluding the lumen. It was associated with abundant material resembling in color and consistency that was found in the abdomen. This neoformation adhered to the lateral walls and fundus of the uterus, extending almost to the cervix (Figure 1B). The ovaries were unscathed.

Histopathologic examination of the mass revealed features of carcinosarcoma consisting almost entirely of a sarcomatous component and small foci of endometrioid- type adenocarcinoma, with neoplastic embolization of the intraparietal venous vessels. An inflammatory infiltrate associated with marked tumor necrosis was also noted (Figures 1C and 1D). With this background, the abdominal findings were interpreted as purulent discharge and necrosis following the adjoining uterine cancer.

At the end of all investigations, the cause of death was identified as massive pulmonary arterial thrombosis in a subject with uterine carcinosarcoma.

For completeness, we also considered peritonitis and sepsis as possible co-participation in the cause of death. However, we considered this hypothesis unlikely based on the macroscopic and microscopic findings. Specifically, the peritoneum, abdominal viscera, and serosa were shiny, without fibrinous deposits, and of normal consistency. The purulent-looking material appeared free and not adherent to the abdominal wall and intestinal loops, as would be the case with recent extravasation, and was not organized. The histologic findings confirmed these considerations, which revealed no evidence of septic embolism. Overall, there were no postmortem findings suggestive of peritonitis and sepsis. Had the massive and acutely fatal pulmonary arterial thrombosis not occurred, local or systemic septic complications most likely could have developed.

The case was considered of interest because of the rare neoplasm, its size, and the very advanced stage of the disease. Another peculiarity was that one of the most lethal direct complications of this neoplasm, pulmonary arterial thrombosis, was observed. Unfortunately, the absence of family members and health information does not allow further clinical considerations, but the severity of the disease found is glaringly the result of neglected and untreated disease. This case demonstrates that such scenarios are still possible today, despite the increased importance of prevention campaigns. In this context, the observed anatomopathological findings are crucial as they enrich the knowledge of uterine carcinosarcoma, and their images may help forensic pathologists who have not previously encountered this rare disease.
